# High-proportions of tailwater discharge alter microbial community composition and assembly in receiving sediments

**DOI:** 10.1038/s41598-024-63859-2

**Published:** 2024-06-19

**Authors:** Yaqian Zhou, Li Cheng, Ying Lian, Jiaying Feng, Mengling Zhou, Dan Jing, Weiwen Yin, Heli Wang, Lei Liu

**Affiliations:** 1 Beijing Institute of Economics and Management, Beijing, 100102 China; 2 School of Water Resources and Environment, China University of Geosciences (Beijing), Beijing, 100083 PR China; 3 China University of Geosciences (Beijing), Beijing, 100083 PR China; 4 Jiangsu Collaborative Innovation Center of Technology and Material of Water Treatment, Suzhou University of Science and Technology, Suzhou, 215009 Jiangsu China; 5 Key Laboratory of Tree Breeding and Cultivation of National Forestry and Grassland Administration, Chinese Academy of Forestry, Research Institute of Forestry, Beijing, 100091 China

**Keywords:** Tailwater, Receiving water, Sediments, Community structure, Co-occurrence network, Microecology, Ecology, Environmental sciences, Hydrology

## Abstract

The tailwater from wastewater treatment plants serves as an important water resource in arid regions, alleviating the conflict between supply and demand. However, the effects of different tailwater discharge proportions on microbial community dynamics remain unclear. In this study, we investigated the effects of different tailwater discharge proportions on the water quality and microbial community characteristics of sediments in receiving water bodies under controlled conditions (WF-1, WF-2, WF-3, WF-4, and WF-5, containing 0% tailwater + 100% natural water, 25% tailwater + 75% natural water, 50% tailwater + 50% natural water, 75% tailwater + 25% natural water, and 100% tailwater + 0% natural water, respectively). Microbial co-occurrence networks and structural equation model were used to unveil the relationship between microbial communities and their shaping factors. Results showed that distinct microbial community compositions were found in the sediments with low- (< 50%) and high- (> 50%) proportions of tailwater. Specifically, *WCHB1-41* and *g_4-29–1*, which are involved in organic degradation-related functions, were the key genera in the high-proportion cluster. A total of 21 taxa were more abundant in the low-proportion (< 50%) cluster than that in high-proportion (> 50%). Moreover, higher modularity was observed in the low-proportion. Total phosphorus directly affected while ammonia nitrogen indirectly affected the microbial community structure. Our findings support the distinct heterogeneity of microbial communities driven by tailwater discharge in receiving water bodies, and further confirmed that high-proportion tailwater depletes sensitive microbial communities, which may be avoided through scientific management.

## Introduction

As a stable supply of alternative water resources, tailwater from wastewater treatment plants (WWTPs) can meet water demands for different purposes. Tailwater is widely used as a source of ecological water replenishment for rivers and lakes. However, as a composite pollution source, tailwater discharge affects water quality and microbial communities in river systems. Systematic assessment studies under controlled conditions are necessary to explore the ecological effects of different tailwater discharge proportions on receiving lake water bodies in Northwest China.

Tailwater has undergone secondary or tertiary treatment to meet the requirements for Class A in the Pollutant Discharge Standard for Municipal Wastewater Treatment Plants (GB 18918–2002). Despite the implementation of multiple levels of biochemical and physicochemical treatment, certain concentrations of nutrients and trace pollutants persist in tailwater. As a result, the direct discharge of tailwater into waterbodies can give rise to several environmental problems and disrupt the ecological function of these bodies^1^. Appropriate amounts of nutrients are essential for maintaining the sustainability of lake ecosystems; however, continuous tailwater discharge can potentially enrich nutrients and significantly alter the physicochemical and biological conditions of the receiving water environment^2^. Still water bodies, such as lakes, are prone to algal blooms and eutrophication caused by tailwater discharge. Tailwater contains various trace pollutants that cannot be completely eliminated by conventional water treatment techniques. After entering the receiving water body, the pollutants in the tailwater are not easily degraded and utilized by microorganisms but are easily enriched in aquatic organisms, entering the food chain and further affecting human health^3^. Research on the distribution and risk of nutrients and trace pollutants in lakes and rivers receiving tailwater discharge has received widespread attention; however, little is known about the ecological effects of tailwater discharge on aquatic organisms, especially microorganisms, in sediments in arid regions^4,5^.

Sediment, an important component of lake ecosystems, serves as a sink for various types of substances^6^. Most pollutants are gradually deposited at the bottom of lakes through natural sedimentation or surface adsorption of particles. These pollutants may also be released into the overlying water body, causing secondary pollution^7–9^, which affects not only the quality of water in the lake but also the bacterial community in the sediment. Microorganisms in sediments maintain the health of lake ecosystems by regulating the flow of energy and the decomposition and transformation of carbon, nitrogen, sulfur, and toxic and harmful pollutants^10,11^. They also contribute to the migration and transformation of organic matter and participate in N cycling, which may help improve the environment of inland saline-alkali lakes^12,13^. Microorganisms in sediments can thus serve as indicators of the overall health of water ecosystems. Therefore, it is crucial to understand the changes in community structure and diversity to elucidate the impact of tailwater discharge and develop effective strategies for lake management and external input control.

In China, arid and semi-arid regions account for about 50 to 55% of the country’s land area, mainly suffering from water scarcity caused by limited water resources. Recycled water, including tailwater, is a valuable water source with great economic value. In recent years, the use of recycled water for ecological replenishment has gradually increased. For example, in Erdos City, Inner Mongolia, the recycling rate of recycled water reached 86% in 2021. The rational use of recycled water is crucial for the ecological environment protection in this region.

In this study, we hypothesize that the discharge of tailwater causes heterogeneity in microbial communities and functions through key microbial groups, and there is a reasonable range for the tailwater discharge proportions. Therefore, we investigated the effects of different tailwater discharge proportions on the bacterial communities in sediments under controlled conditions by conducting a laboratory lake microcosmic experiment^14^. The specific objectives of this study were to (1) clarify the diversity and structural differences in bacterial communities under different tailwater discharge proportions, (2) reveal the main environmental factors driving changes in bacterial communities through structural equation modeling, and (3) elucidate the interactions between bacterial communities and their response to environmental changes through co-occurrence networks. This study provides an important scientific basis for water pollution control and water replenishment in arid and semiarid lakes.

## Materials and methods

### Sampling point setting

The tailwater samples were collected from the local WWTP (109° 48′ E, 39° 32′ N) in Ejin Horo Banner (Ordos) (109° 40′ E, 39° 17′ N, 109° 41′ E), Inner Mongolia, China, which is a typical arid and semiarid region. This plant is a typical representative of municipal sewage plants in northern China in terms of sewage source, treatment process, and operation management. The main treatment processes included primary physical separation (grid and primary sedimentation tank), modified Bardenpho biological treatment, and coagulation precipitation and ultrafiltration treatment. The effluent met the criteria for Class A in the Cities Sewage Treatment Plant Pollutant Discharge Standard (GB 18918-2002). Natural water and sediment were obtained from the unpolluted branch lakes of the Ulam Mulun River (109° 41′ E, 39° 38′ N), which is a secondary tributary of the Yellow River and whose water quality meets the criteria for Class III of the national surface water quality standard (GB 3838-2002) in China. The water samples consisted of the WWTP effluent mixed with uncontaminated natural water.

### Experimental setting

The organisms used in this experiment were submerged plants, aquatic animals, and microbial communities collected from natural water sampling sites. To ensure stabilization and colonization, the collected natural water, sediment, submerged plants (*Myriophyllum verticillatum*), and aquatic animals (including zebrafishs, black shell shrimps and apple snails) were inoculated together in a 126 L plastic cuboid container (70 cm × 45 cm × 40 cm) for a 2-week acclimatization period. Only aquatic organisms with strong adaptability were selected for the experiment.

**Figure 1:**
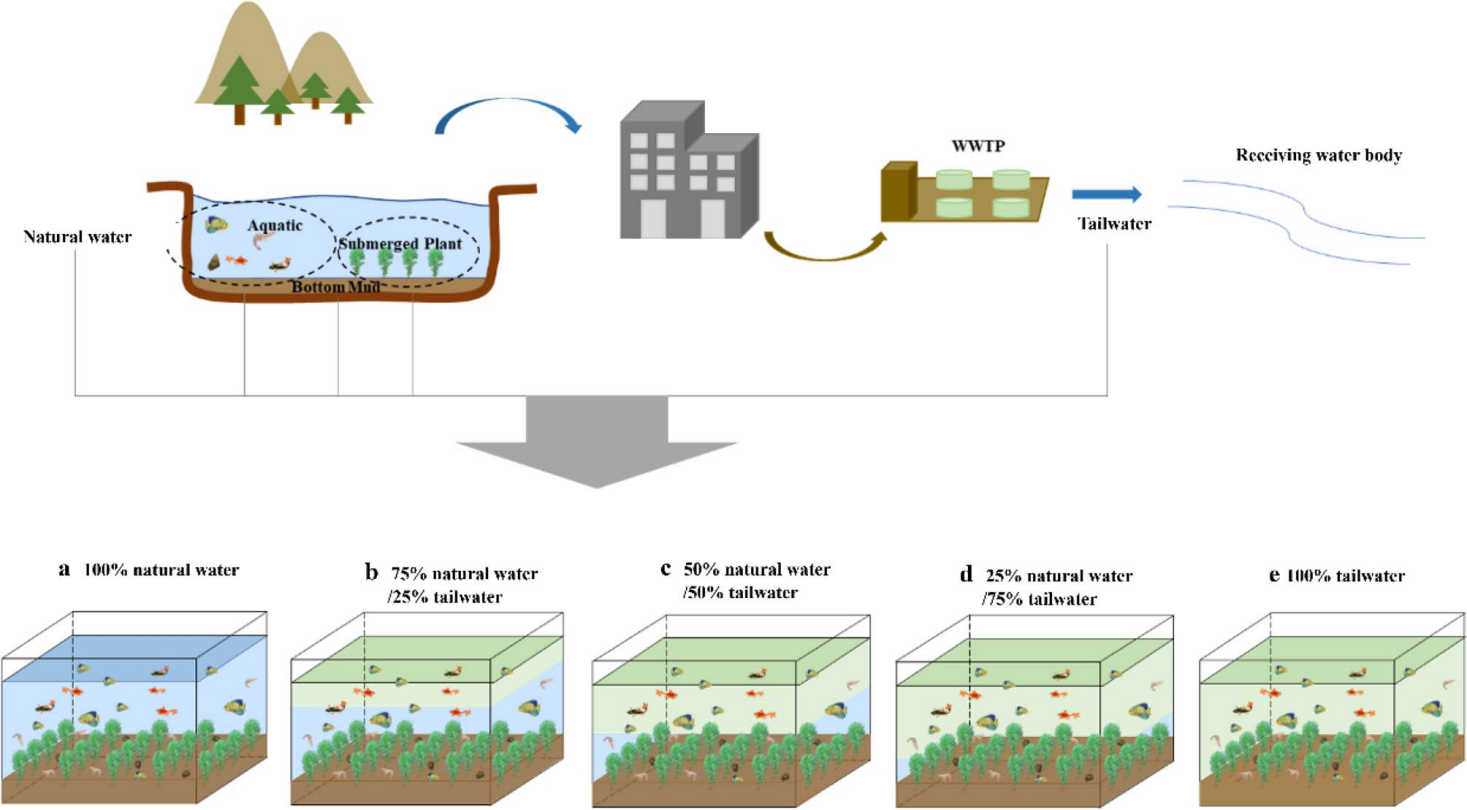
Experimental setup: (**a**) 100% natural water, (**b**) 75% natural water/25% tailwater, (**c**) 50% natural water/50% tailwater, (**d**) 25% natural water/75% tailwater, (**e**) 100% tailwater.

A natural lake ecosystem was simulated in a 34.8 L polyethylene box (40 cm × 29 cm × 30 cm). A 5 cm thick layer of the stabilized sediment was placed at the bottom of the box. The submerged plants were evenly transplanted into the sediment at a density of 20 plants/box. The tailwater and natural water were mixed in different proportions using the siphon method, with WF-1, WF-2, WF-3, WF-4, and WF-5 representing mixtures containing 0, 25, 50, 75, and 100% tailwater (Fig. 1). Each treatment was replicated thrice to ensure reproducibility. The physicochemical characteristics of the unpolluted natural surface water and tailwater are listed in Table S1.

All experiments were conducted under a 12 h light/12 h dark cycle and a constant temperature of 25° C^15,16^. After 2 days of water stabilization and clarification, aquatic animals (shrimp, snails, and fish) were placed in the simulation box for 45 days. For oxygen exchange, all treatments were in an open environment. On days 1 and 45, water samples (50 mL) were filtered through a glass fiber filter membrane with a pore size of 0.22 μm and used in hydrochemical detection. On day 45, sediment samples from the top layer (0–2 cm) at the bottom of 15 simulated boxes were collected using the five-point sampling method and then stored in sterilized polypropylene tubes at −80° C to analyze bacterial community responses^16^.

### Water quality test method

The levels of chemical oxygen demand chromium (COD_Cr_), ammonia nitrogen (NH_4_^+^–N), and total phosphorus (TP) were determined using the Detection and Analysis Method for Water and Wastewater (4th edition) compiled by the China Environmental Protection Administration. COD was determined through rapid digestion and spectrophotometry: a small amount of water sample was digested at 120° C for 2 h in a sealed reagent tube using a digester (LB-901A, Qingdao Lubo Jianye Environmental Protection Technology Co., Ltd., Shandong, China) and determined through spectrophotometry. NH_4_^+^-N content was determined using salicylic acid-hypochlorite spectrophotometry, and the content of the blue compound formed by the reaction of ammonium and hypochlorite ions was measured at 697 nm using a UV spectrophotometer (UV-1900i, NMIE, USA). TP content was determined using molybdate-antimony-scandium spectrophotometry.

### Detection of microbial community in sediments

Total genomic DNA of the microbial community was extracted from 0.5 g sediment samples using the Soil Rapid DNA Rotation Kit (MP Biomedical, Solon, OH, USA). After the purity, concentration, and integrity of the DNA were tested, the V3–V4 hypervariable region of the 16S rRNA gene was amplified on GeneAmp PCR System 9700 (ABI Inc., USA) using primers 338F (5′-ACTCCTACGGGAGGCAGCAG-3′) and 806R (5′-GGACTACHVGGGTWTCTAAT-3ʹ).

PCR products from the same sample were detected and identified using 2% agarose gel electrophoresis. The PCR products were purified and quantified using AxyPrep DNA gel extraction kit (AxyPrep DNA Gel Extraction Kit, Union City, CA, USA) and Quantus™ Fluorometer (Promega, USA), respectively. A DNA Gel Extraction Kit (Axygen, Union City, CA, USA) was used to construct a database of PCR products (~ 100 ng). The PCR products were sequenced on a MiSeq PE300 platform (Illumina, San Diego, CA, USA).

Fastp v. 0.20.0 (https://github.com/OpenGene/fastp) and FLASH v. 1.2.7 (http://www.cbcb.umd.edu/software/flash) were used to control the quality of the original sequence and read the content of merger and stitching, respectively. Based on 97% similarity, operational classification units (OTUs) were clustered using UPARSE version 7.1, and the corresponding OTU table was generated. According to the Silva_v138 16S rRNA database (http://www.arb-silva.de), species classification annotation was performed for each OTU representative sequence using the RDP Classifier v.2.2, with a comparison threshold of 0.7.

### Statistical analysis

The different responses of microorganisms to environmental changes were further studied using QIIME (https://qiime2.org) or R package v. 3.3. 1 (R Core Team, Vienna, Austria). The alpha diversity indices of the samples were analyzed using Statistical Product and Service Solutions (SPSS), and their significance levels were compared using Analysis of Variance (ANOVA). Origin v. 2018 was used to describe the species composition and abundance of the microbial community with different color gradients based on gene classification results. Nonmetric multidimensional scale (NMDS) analysis based on the Bray–Curtis distance was conducted to explain differences in bacterial communities. The co-occurrence network was calculated based on the Spearman rank correlation coefficient to explore the interactions between bacterial communities. Spearman correlation coefficients were calculated using the R package “psych.” The network diagram was visualized using Gephi 0.9.1. The effects of important environmental factors on other factors and communities were visualized through structural equation modeling (SEM) using IBM SPSS AMOS 23.0.

## Results and discussion

### Changes in water chemistry

Table 1 presents the water chemistry of the different treatments. The results showed no significant difference in pH and dissolved oxygen (DO) among the treatments (*p* > 0.05), indicating that the addition of tailwater did not significantly affect the DO and pH in the ecosystem under controlled conditions in the small-scale experiment. At the end of the experiment, the levels of COD_Cr_, TP, ammonia nitrogen, and total nitrogen showed an overall upward trend as tailwater discharge proportions was increased. This trend indicates that an increase in tailwater discharge proportions may increase the nutrient load of the receiving water body, which is related to the initial value of the added tailwater^17^. Therefore, direct discharge to a receiving lake may cause significant ecological problems, such as eutrophication^18–20^. Therefore, this type of sewage plant must be urgently upgraded and renovated. In addition, further research is needed to investigate its impact on the water quality and microbial communities of receiving lakes, develop reasonable management measures, and reduce ecological risks.

**Table 1:** Water quality of different treatments.

Index	Treatment				
	WF-1	WF-2	WF-3	WF-4	WF-5
pH	8.50 ± 0.12	8.92 ± 0.21	8.39 ± 0.12	9.03 ± 0.03	8.52 ± 0.10
Chemical oxygen demand (COD, mg/L)	44.23 ± 0.33	42.98 ± 0.22	56.33 ± 0.42	53.41 ± 0.14	50.24 ± 0.58
Phosphate (mg/L)	0.19 ± 0.01	0.20 ± 0.03	0.25 ± 0.02	0.28 ± 0.02	0.24 ± 0.03
Total nitrogen (mg/L)	4.61 ± 0.04	4.13 ± 0.02	4.93 ± 0.07	4.82 ± 0.03	5.39 ± 0.02
Ammonia nitrogen (mg/L)	0.06 ± 0.02	0.05 ± 0.01	0.07 ± 0.03	0.08 ± 0.02	0.08 ± 0.01

### Variation of the microbial community in sediments

**Figure 2:**
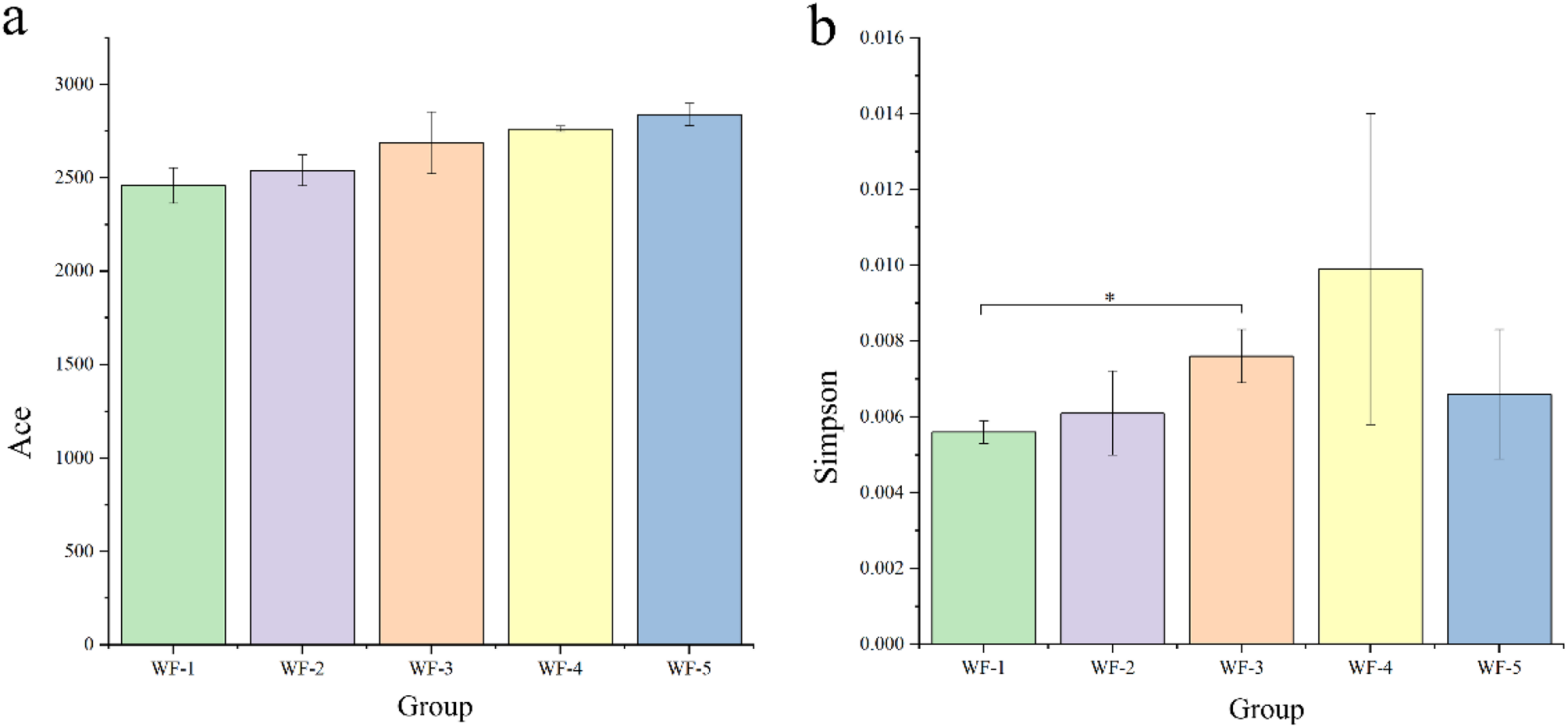
Alpha indices of the five experimental groups at the OTU level. (**a**) Ace index, (**b**) Simpson index. Analysis of variance (ANOVA) was used to test significant differences in index values between two groups. *indicates significant differences (*p < 0.05, **p < 0.01, ***p < 0.001).

A total of 517,497 optimized sequences were generated from 10 samples through high-throughput sequencing of the 16S rRNA gene. The average trimmed sequence was 414 bp. The coverage index of each treatment was above 0.97 (Table S2), indicating that the sequencing library reflected the actual bacterial community in the sediments. The results of α diversity analysis indicated that the Simpson index showed no significant difference among the treatments (*p* > 0.05; Fig. 2a). However, the Ace index showed an overall upward trend as the tailwater gradient increased (Fig. 2b). Moreover, the Ace index values for WF-1 and WF-2 differed significantly from those for WF-3, WF-4, and WF-5 (*p* < 0.05; Table S2). These results suggest that tailwater discharge affects the microbial community in the receiving water^21^ and there was a threshold range in the mixing process that may have significant a impact on the microbial communities.

**Figure 3:**
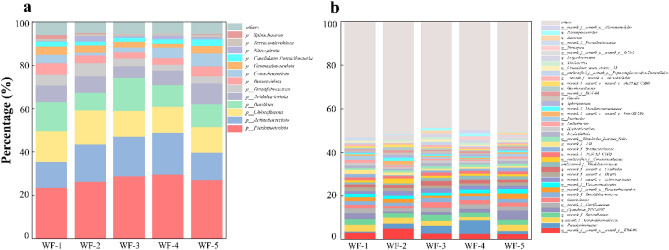
Taxonomic maps of different groups of microorganisms at the phylum (**a**) and genus (**b**) levels. Only taxa with abundance > 1.0% are shown, while those with abundance < 1.0% are represented by “Others.” Different colors represent different taxa.

The effects of different tailwater discharge proportions on the composition and structure of microbial communities were further analyzed. In this study, 52 phyla, 148 classes, 353 orders, 581 families, 1052 genera, 1896 species, and 4107 OTUs were obtained. At the phylum level, each treatment shared the same dominant phylum; however, the abundance of the dominant phyla varied among the treatments (Fig. 3a). Twelve phyla, including *Pseudomonadota*, *Actinobacteriota*, *Chloroflexota*, *Bacillota*, *Acidobacteriota*, and *Cyanobacteriota*, had a relative abundance > 1%. *Pseudomonadota* and *Actinobacteriota* were the dominant phyla in sediments. They are considered to play a crucial role in the conversion of diverse substances, including carbon, nitrogen, and phosphorus^22^. *Bacillota* is widely present in various environments and can dominate low organic load environments^23^. *Chloroflexota*, which is distributed in various habitats, has multiple nutritional and metabolic pathways and participates in important biogeochemical cycles, such as those of C, N, P, and S^24^.

At the genus level, 37 genera had a relative abundance greater than 1%, of which only 40.5% were cultivable bacterial treatments (Fig. 3b). The genus with the highest relative abundance was *KD4-96*, which was lowest in WF-1 (control) (2.52%) than other in the treatments (WF-2, WF-3, WF-4 and WF-5). As the proportions of tailwater was increased, the highest relative abundance of *KD4-96* was 5.01% (WF-4), followed by 3.21% (WF-5). Previous studies have shown that *KD4-96* has a strong ability to metabolize recalcitrant organic compounds; therefore, enrichment of *KD4-96* may improve the mineralization of organic compounds. The genus with the second highest abundance was *Pseudarthrobacter*, which initially increased and then decreased with increasing tailwater discharge proportions. The highest and lowest abundance of *Pseudarthrobacter* occurred in WF-2 (6.13%) and WF-5 (0.47%), respectively. *Pseudarthrobacter* is a typical denitrifying phosphorus-accumulating organisms (DPAOs) that can complete nitrogen and phosphorus removal in the same strain and under the same condition, thereby improving the efficiency of biological nitrogen and phosphorus removal in aquatic ecosystems^25^. We speculate that the enrichment of this genus may influence the nitrogen and phosphorus concentrations in ecosystems^26,27^. The genus with the third highest relative abundance was *norank_f_Gemmatimonadaceae*, which includes typical denitrifying bacteria that participate in the nitrogen cycle of anaerobic ammonia oxidation as a core group^25^. In contrast to that of *Pseudomonas*, the relative abundance of *norank_f_Gemmatimonadaceae* was the lowest in WF-2 (1.52%) and it peaked in WF-5 (3.09%) as the tailwater discharge proportions was increased. These results suggest that the succession of bacterial communities is closely related to the tailwater discharge proportions^26,28^.

### β diversity analysis of bacterial communities

**Figure 4:**
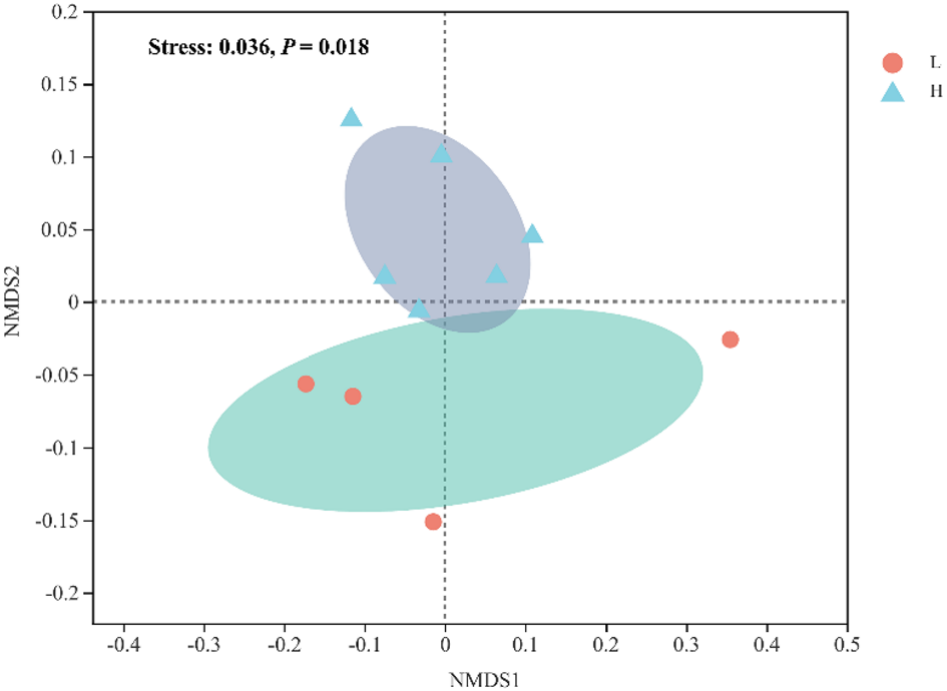
Nonmetric multidimensional scaling (NMDS) of microbial community composition based on Bray–Curtis differences between five groups of simulation experiments. Stress < 0.20 is more statistically significant.

NMDS analysis based on the Bray–Curtis distance was conducted to analyze and compare the bacterial communities at the OTU level among the different treatments. As shown in Fig. 4, the five treatments (WF-1, WF-2, WF-3, WF-4, and WF-55) were statistically significantly clustered into low-proportion (L, WF-1, and WF-2) and high-proportion (H, WF-3, WF-4, and WF-5) (*p* < 0.05) treatments. This clustering result indicated that the microbial communities underwent significant changes between WF-2 and WF-3 (tailwater discharge proportions in the range of 25–50%). These results are consistent with previous study, which suggested that the microbial community structure in sediments undergoes significant changes when the effluent of sewage treatment plants accounts for approximately 50% of the total river flow^14^.

**Figure 5:**
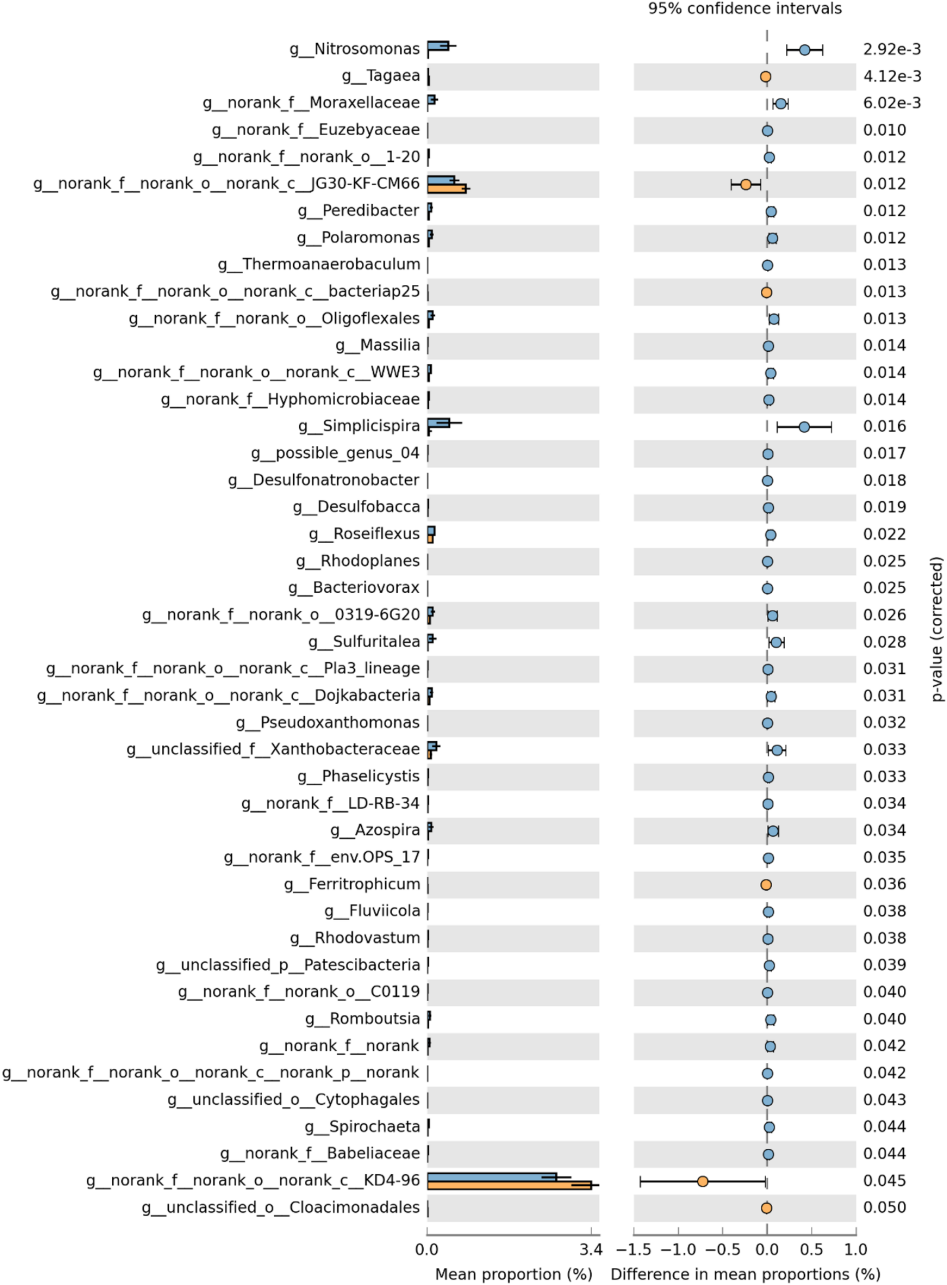
Significant differences in bacterial community at the genus level between the high- and low-proportion clusters, where blue represents the high-proportion cluster, orange represents the low-proportion cluster. The left-hand side shows the classification with significant differences in species composition between clusters and the proportion in each cluster, and the right-hand side shows the proportion, confidence interval, and *p*-value of the difference.

The Wilcoxon rank-sum test was conducted to test the significance of the bacterial communities between the two clusters at the genus level (Fig. 5). The relative abundance of *Nitrosomonas* significantly differed between the two clusters (*p* < 0.001).*Nitrosomonas* is an important component of ammonia-oxidizing bacteria, and its abundance can rapidly increase from less than 1 to 7% with increasing ammonia-nitrogen concentrations^29^;. In the present study, the abundance of *Nitrosomonas* in the high-proportion cluster (0.4%) was 20 times higher than that in the low-proportion cluster (0.02%). This result indicates that the discharge of tail water affects the concentration of ammonia nitrogen in the receiving water, thereby affecting the main functional microorganisms in nitrogen cycling, which is consistent with the change in ammonium ion concentration in hydrochemical indicators^30–32^. Although the relative abundance was relatively low, there were significant differences in the relative abundance of *g-Taeaga*, *g-Moraxellaceae*, and *g-Euzebyaceae* between the low- (< 50%) and high- (> 50%) clusters. Previous studies have suggested that all three genera are involved in biological processes, such as nitrate reduction and phosphate dissolution. Overall, we found significant differences in 44 bacterial genera (*p* < 0.05), with only 6 genera having higher abundances in the low-proportion cluster and the 38 other genera significantly increasing in the high-proportion cluster. All 44 genera with significant differences had relative abundances less than 1%, indicating that rare bacteria exert an important influence on the microbial community structure. The increase in tailwater discharge proportions had a profound influence on microbial reproduction and succession, ultimately resulting in an augmentation of the rare microbial communities.

### The influence of increased tailwater discharge proportions on the interaction between bacteria in sediments

**Figure 6:**
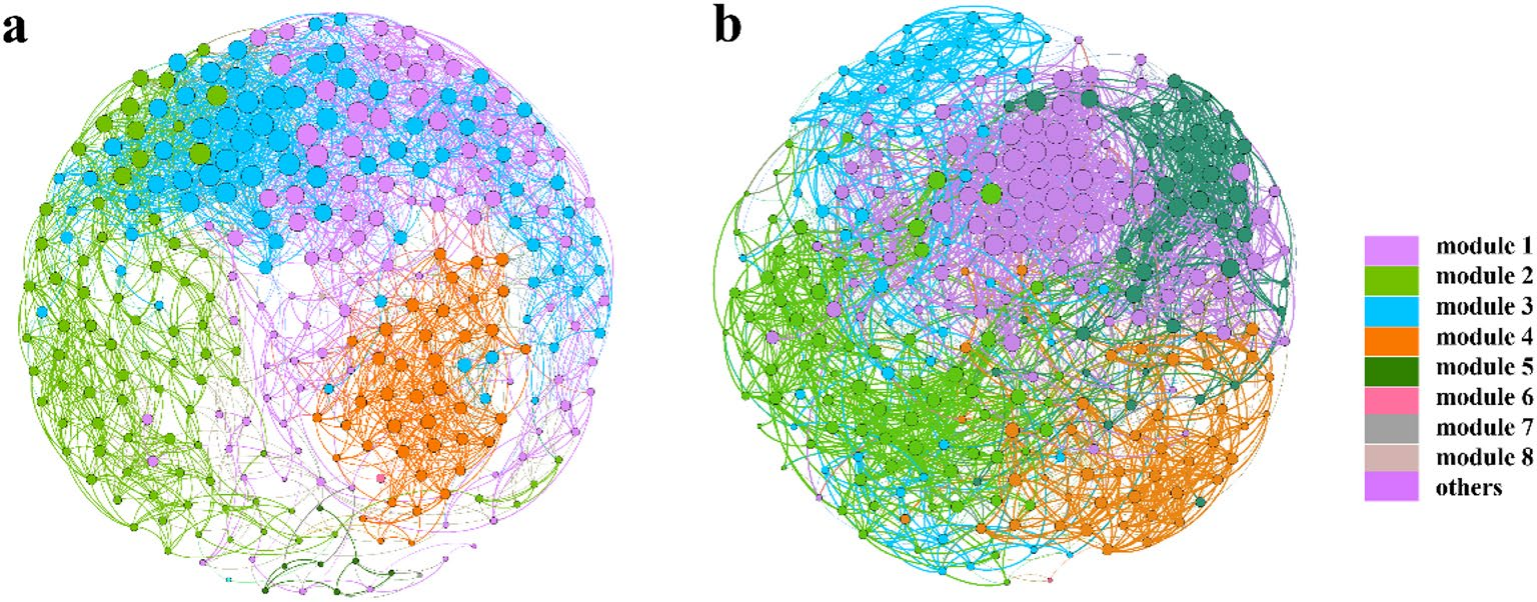
Microbial networks in two proportion clusters. Co-occurrence networks of the bacterial communities at the genus level are colored by modularity class in (**a**) low and (**b**) high proportions. Positive and negative correlations are labeled with red and blue edges, respectively.

The interactions among microorganisms reflect the structure of microbial communities, and exploring this relationship is beneficial for elucidating the succession patterns of complex microbial communities across tailwater discharge proportions^33,34^. A co-occurrence network was constructed to explore the interaction patterns of microbial communities under low- (< 50%) and high- (> 50%) proportion of the effluent (Fig. 6).

**Figure 7:**
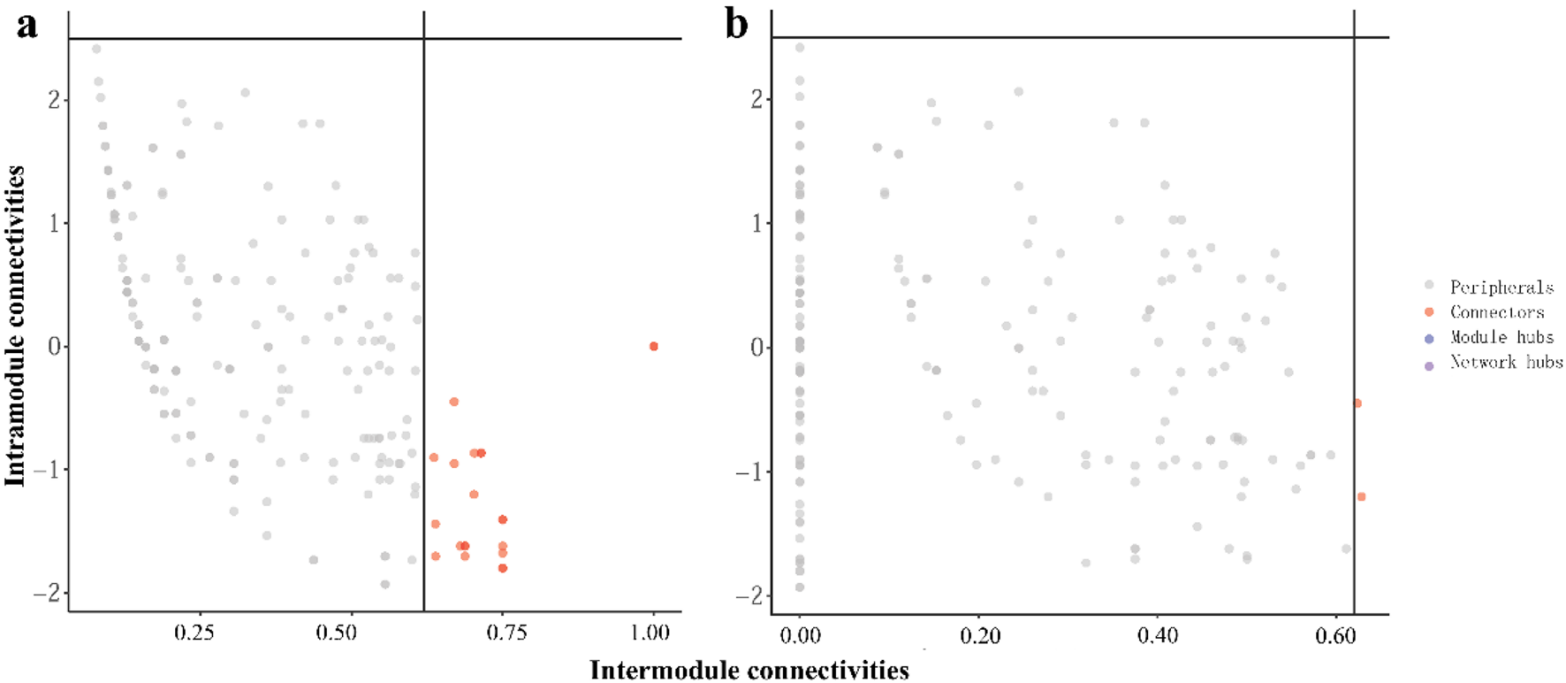
Zi-Pi plots highlighted the key genus within the microbial network of the low- (**a**) and high- (**b**) proportions clusters.

Overall, the modularity levels of the low- and high-proportion clusters were significantly higher than 0.65, indicating strong interactions between species within the two clusters. Furthermore, the edge number, average degree, modularity number, and modularity level in the low-proportion cluster were significantly higher than those in the high-proportion cluster (*p* < 0.05), indicating that the inter-species interactions in the low-proportion cluster were more complex than those in the high-proportion cluster. We categorized the nodes separately based on the intro module (Zi) and intermodal (Pi) connections within the two clusters, taking into consideration the network topology characteristics and the various ecological roles of the nodes^35,36^ (Fig. 7). The majority of the two clusters belonged to peripheral nodes, indicating that most of them were connected within the module, with fewer connections to nodes in other modules^37^. No module or network center was identified in either the low- or high-proportion cluster. The low-proportion cluster had 21 connection nodes, whereas the high-proportion cluster had only 2 connection nodes. The number of connection nodes in the high-proportion cluster was substantially lower than that in the low-proportion cluster. In general, connection nodes can be highly connected to multiple modules, which is crucial for maintaining network connectivity and community stability, and the connection nodes are defined as key species^35,38^. Therefore, based on the topological characteristics of the networks and the distribution of key species, we speculated that the microbial communities of the high-proportion cluster were significantly influenced by tailwater discharge. Consequently, key groups either underwent extinction or experienced succession to adapt to environmental changes, potentially compromising ecosystem stability.

Further analysis of the key microbial groups revealed that *WCHB1-41* and *g_4-29-1* were the key genera in the high-proportion cluster (Table S3). Bacteria belonging to the genus *WCHB1-41* promote the degradation of organic matter, such as long-chain fatty acids, and rapidly reproduce and accelerate substrate methanation under anaerobic conditions^39^ (Table S3). In addition, we did not find any relevant reports on *g_4-29-1* in the database, indicating that this genus might be uncultivatable. Similarly, in the low-proportion cluster, 8 out of 21 key genera had no relevant information and thus may be uncultivatable (Table S3). However, the 13 other genera played dominant roles in water quality purification, including participating in organic matter metabolism, participating in nitrogen fixation and denitrification, and driving phosphorus differentiation and transformation. Among these genera, *g_Erysipelothrix* is potentially pathogenic^40^; *g__CL500-29_ Marine_Group* plays a crucial role in water quality purification^41^; *g_Altererythrobacter* is key genera involved in the DNRA process^42^, *g_Caulobacter* is closely related to the biological metabolism of phosphorus in soil^43^; *g_Lysobacter* drives phosphorus differentiation and transformation, and its abundance is positively correlated with phosphorus content^43^; and *g_Peredibacter* is key genera usually involved in the metabolism of recalcitrant organic compounds.

In this study, the key genus exhibited considerably higher abundance in the low-proportion cluster than it did in the high-proportion cluster. This disparity could be attributed to the substantial influence of the tailwater discharge proportion on the receiving water body after surpassing a reasonable threshold range. Consequently, the water body may transition into an anaerobic state. Furthermore, from the perspective of microbial community functions, the main role of the key genus in the low-proportion cluster is the purification of water and maintenance of ecosystem health through the coordination of organic matter decomposition, nitrogen cycling, and phosphorus cycling, which indicated that environmental changes within a reasonable range of tailwater discharge proportion could enhance the proliferation of functional microorganisms, thereby enabling resistance to external disturbances^44^.

### Factors shaping the sediment microbial community under different tailwater discharge proportions

**Figure 8:**
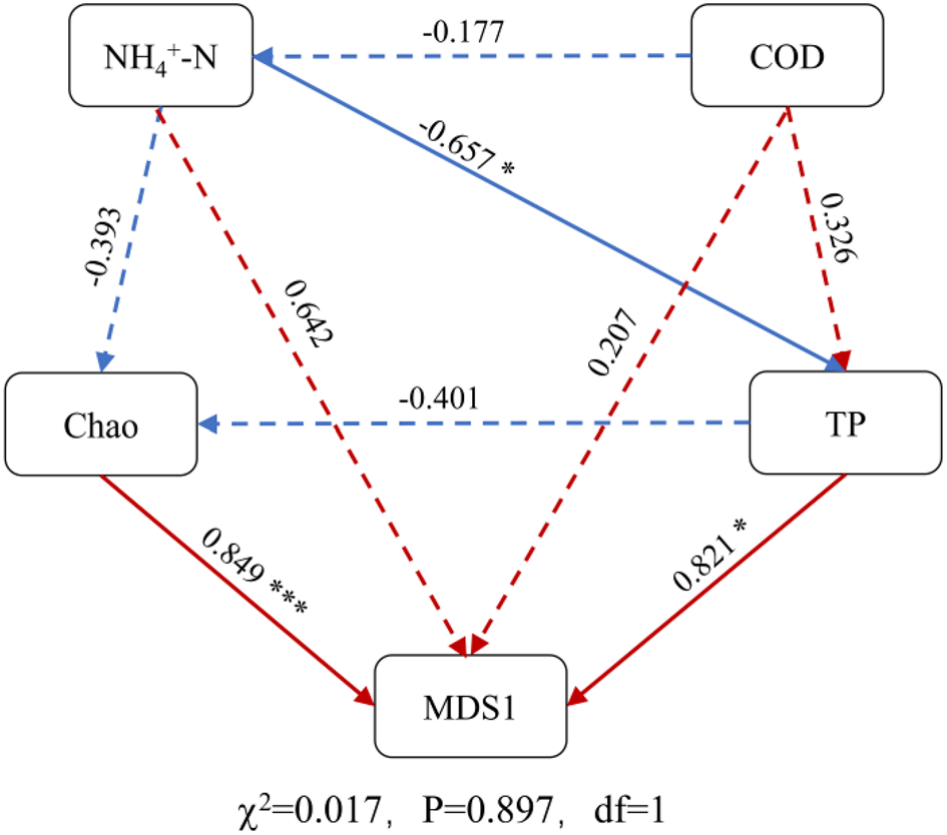
Structural equation model showing direct and indirect effects of key factors on microbial community composition. Red and blue lines indicate positive and negative pathways, respectively. The standardized path coefficients are next to the arrows. *indicates significance levels: *p < 0.05, **p < 0.01, ***p < 0.001.

SEM analysis was conducted to investigate the relationship between typical environmental factors, diversity indices, and microbial communities in the low- and high-proportion clusters and to clarify the factors influencing microbial community changes (Fig. 8). The Chao1 index was significantly correlated with microbial communities (λ = 0.849, *p* < 0.001). This result is consistent with the previous finding that tailwater discharge proportion is primarily responsible for the richness of microbial communities^45,46^. TP directly affected the composition of microbial communities (λ = 0.821, *p* < 0.05) and showed a significant negative correlation with ammonia nitrogen (λ =−0.657, *p* < 0.05), suggesting that ammonia nitrogen indirectly affected the bacterial communities.

The discharge of tailwater from domestic sewage treatment plants leads to the accumulation of nutrients, such as nitrogen and phosphorus. Nitrogen and phosphorus, which are characteristic pollutants of eutrophication in water bodies, may cause the rapid reproduction of algae and other plankton and disrupt the balance of the ecosystem^47^. The nitrogen and phosphorus cycles have a coupled effect. Organic matter in receiving waters usually accumulates on the sediment surface. The mineralization and decomposition of organic matter leads to an anaerobic state, resulting in the release of phosphorus. The enrichment of phosphorus can change the composition of bacterial communities in water and increase the abundance of denitrifying bacteria, thereby promoting denitrification^48^. Moreover, among bacteria closely related to nitrogen and phosphorus, DPAOs have attracted considerable attention because of their ability to simultaneously remove nitrogen and phosphorus^49,50^. In the present study, we identified a DPAO, *Pseudarthrobacter*, whose abundance was highest in WF-2 and gradually decreased with increasing tailwater discharge proportions. *Pseudarthrobacter* actively participated in the nitrogen and phosphorus removal in WF-2, which was consistent with the nitrogen and phosphorus concentrations in the water chemistry data of WF-2 (ammonia nitrogen, TP, and total nitrogen had the lowest values in each treatment). This speculation further supports the significant correlation between nitrogen and phosphorus in the SEM^51^. The finding that *Pseudarthrobacter* had the highest abundance in WF-2 was consistent with the clustering results for high- and low- proportion, which further verified the impact of tailwater discharge proportions on the microbial community.

In general, the discharge of tailwater affects typical environmental factors, particularly the TP and ammonia nitrogen contents, in the receiving water body, which further impacts the succession of microbial communities in the receiving water body and the physicochemical and biological processes in the ecosystem^37,52^. Future in-depth work will use metagenomics to illustrate changes in the abundance of functional genes involved in geochemical cycles, such as carbon, nitrogen, phosphorus, and sulfur.

## Conclusion

The discharge of high-proportion tailwater caused the accumulation of nitrogen, phosphorus, and other nutrients in the receiving water body, which had a significant impact on the diversity of bacterial communities. Total phosphorus was an important factor in the changes of bacterial communities caused by tailwater discharge. It is suggested that the treatment standards of sewage treatment plants should be further improved in arid areas, especially for the control of total phosphorus and ammonia nitrogen. Under different proportions of tailwater discharge, the diversity of bacterial communities exhibited a non-linear trend. Key species undergo succession processes to adapt to environmental changes, which in turn affects the stability of the ecosystem. In the high-proportion cluster, *WCHB1-41* and *g_4-29-1*, which were involved in organic degradation-related functions, were the key genera. In the low-proportion cluster, about 21 key genera mainly participated in biogeochemical processes, such as those involving organic matter, nitrogen, and phosphorus cycling, through coordinated communities. Overall, tailwater discharge may affect complex physicochemical and biochemical processes in the receiving water body. The resulting response patterns of microbial communities and changes in key taxa may have an impact on ecosystem stability. Therefore, it is necessary to control the proportion of tailwater discharge and ensure that the concentration does not exceed 50% in arid regions.

### Supplementary Information

Supplementary Tables.

## Data Availability

All data generated or analysed during this study are included in this published article (and its Supplementary Information files).

## Abbreviations

*WWTP*: Wastewater treatment plant
*COD*_*Cr*_: Chemical oxygen demand chromium
*NH*_*4*_^+^*–N*: Ammonium nitrogen
*TP*: Total phosphorus
*OUT*: Operational classification unit
*NMDS*: Nonmetric multidimensional scale
*SEM*: Structural equation modeling

## References

[1] Carey RO, Migliaccio KW (2009). Contribution of wastewater treatment plant effluents to nutrient dynamics in aquatic systems: A review. Environ. Manage..

[2] Venkatesh G, Brattebø H (2011). Environmental impact analysis of chemicals and energy consumption in wastewater treatment plants: Case study of Oslo Norway. Water Sci. Technol..

[3] Ezechiáš M, Covino S, Cajthaml T (2014). Ecotoxicity and biodegradability of new brominated flame retardants: A review. Ecotoxicol. Environ. Saf..

[4] Huo Y, Bai Y, Qu J (2017). Unravelling riverine microbial communities under wastewater treatment plant effluent discharge in large urban areas. Appl. Microbiol. Biotechnol..

[5] Olano H, Martigani F, Somma A, Aubriot L (2019). Wastewater discharge with phytoplankton may favor cyanobacterial development in the main drinking water supply river in Uruguay. Environ. Monit. Assess..

[6] Liu Q, Wu H, Huang C, Lin C, Li W, Zhao XF, Li Z, Lv S (2022). Microbial compositions, ecological networks, and metabolomics in sediments of black-odour water in Dongguan. Ch. Environ. Res..

[7] Zhang J, Buyang S, Yi Q, Deng P, Huang W, Chen C, Shi W (2023). Connecting sources, fractions and algal availability of sediment phosphorus in shallow lakes: An approach to the criteria for sediment phosphorus concentrations. J. Environ. Sci..

[8] Woldemedhin DG, Gemeda FT, Abdissa B, Guta DD, Tefera T, Senbeta F (2021). Determinants of people's willingness to pay to restore polluted urban rivers: The case of River Kebena. Groundw. Sustain. Dev..

[9] Feng W, Gao J, Wei Y, Liu D, Yang F, Zhang Q, Bai Y (2022). Pattern changes of micobial communities in urban river affected by anthropogenic activities and their environmental driving mechanisms. Sci. Eur. Environ..

[10] Gerbersdorf SU, Hollert H, Brinkmann M, Wieprecht S, Schüttrumpf H, Manz W (2011). Anthropogenic pollutants affect ecosystem services of freshwater sediments: The need for a ‘triad plus x’ approach. J. Soils Sediments.

[11] Qin B, Zhou J, Elser JJ, Gardner WS, Deng J, Brookes JD (2020). Water depth underpins the relative roles and fates of nitrogen and phosphorus in lakes. Environ. Sci. Technol..

[12] Gao J, Feng W, Yang F, Liu J, Fan W, Wang Y, Zhang Q, Yang W (2022). Effects of water quality and bacterial community composition on dissolved organic matter structure in Daihai lake and the mechanisms. Environ. Res..

[13] Saarenheimo J, Aalto SL, Rissanen AJ, Tiirola M (2017). Microbial community response on wastewater discharge in boreal lake sediments. Front. Microbiol..

[14] Romero F, Sabater S, Font C, Balcázar JL, Acuña V (2019). Desiccation events change the microbial response to gradients of wastewater effluent pollution. Water Res..

[15] Ruprecht J, Birrer S, Dafforn K, Mitrovic S, Crane S, Johnston E, Wemheuer F, Navarro A, Harrison A, Turner I, Glamore W (2021). Wastewater effluents cause microbial community shifts and change trophic status. Water Res..

[16] Zhou Y, Lian Y, Liu T, Jin X, Wang Z, Liu X, Zhou M, Jing D, Yin W, Feng J, Wang H, Zhang D (2023). Impacts of high-quality coal mine drainage recycling for replenishment of aquatic ecosystems in arid regions of China: Bacterial community responses. Environ. Res..

[17] Link M, von der Ohe P, Voß K, Schäfer R (2017). Comparison of dilution factors for German wastewater treatment plant effluents in receiving streams to the fixed dilution factor from chemical risk assessment. Sci. Total Environ..

[18] Ekka S, Haggard B, Matlock M, Chaubey I (2006). Dissolved phosphorus concentrations and sediment interactions in effluen-dominated ozark streams. Ecol. Eng..

[19] Song K, Adams C, Burgin A (2017). Relative importance of external and internal phosphorus loadings on affecting lake water quality in agricultural landscapes. Ecol. Eng..

[20] Wang J, Chen J, Yu P, Yang X, Zhang L, Geng Z, He K (2020). Oxygenation and synchronous control of nitrogen and phosphorus release at the sediment-water interface using oxygen nano-bubble modified material. Sci. Total Environ..

[21] Martínez-Santos M, Lanzén A, Unda-Calvo J, Martín I, Garbisu C, Ruiz-Romera E (2018). Links between data on chemical and biological quality parameters in wastewater-impacted river sediment and water samples. Data Brief..

[22] Guo Y, Wu C, Wang Z, Shi Y, Sun J (2023). Co-occurrence of toxic metals, bacterial communities and metal resistance genes in coastal sediments from Bohai bay. Environmental Pollution.

[23] He W, Chen M, Schlautman MA, Hur J (2016). Dynamic exchanges between DOM and POM pools in coastal and inland aquatic ecosystems: A review. Sci. Total Environ..

[24] Ke S, Xiao Y, Weiss ST, Chen X, Kelly CP, Liu YY (2023). A computational method to dissect colonization resistance of the gut microbiota against pathogens. Cell Rep. Methods.

[25] Eo J, Park K-C (2016). Long-term effects of imbalanced fertilization on the composition and diversity of soil bacterial community. Agric. Ecosyst. Environ..

[26] Lamba J, Anand S, Dutta J, Chatterjee S, Nagar S, Celin SM, Rai PK (2021). Study on aerobic degradation of 2,4,6-trinitrotoluene (TNT) using pseudarthrobacter chlorophenolicus collected from the contaminated site. Environ. Monit. Assess..

[27] Jia L, Cheng X, Fang L, Huang X (2023). Nitrogen removal in improved subsurface wastewater infiltration system: Mechanism, microbial indicators and the limitation of phosphorus. J. Environ. Manage..

[28] Naeimi M, Shavandi M, Alaie E (2021). Determining the impact of biofilm in the bioaugmentation process of benzene-contaminated resources. J. Environ. Chem. Eng..

[29] Atashgahi S, Aydin R, Dimitrov MR, Sipkema D, Hamonts K, Lahti L, Maphosa F, Kruse T, Saccenti E, Springael D, Dejonghe W, Smidt H (2015). Impact of a wastewater treatment plant on microbial community composition and function in a hyporheic zone of a eutrophic river. Sci. Rep..

[30] Silyn-Roberts G, Lewis G (2001). In situ analysis of *Nitrosomonas spp.* in wastewater treatment wetland biofilms. Water Res..

[31] Liu Y, Wei D, Xu W, Feng R, Du B, Wei Q (2019). Nitrogen removal in a combined aerobic granular sludge and solid-phase biological denitrification system: System evaluation and community structure. Bioresour. Technol..

[32] Siddiqi MZ, Sok W, Choi G, Kim SY, Wee JH, Im WT (2020). *Simplicispira hankyongi sp.* nov., a novel denitrifying bacterium isolated from sludge. Antonie Leeuwenhoek.

[33] She Z, Pan X, Wang J, Shao R, Wang G, Wang S, Yue Z (2021). Vertical environmental gradient drives prokaryotic microbial community assembly and species coexistence in a stratified acid mine drainage lake. Water Res..

[34] Niu L, Li Y, Li Y, Hu Q, Wang C, Hu J, Zhang W, Wang L, Zhang C, Zhang H (2021). New insights into the vertical distribution and microbial degradation of microplastics in urban river sediments. Water Res..

[35] Guimerà R, Sales-Pardo M, Amaral LAN (2006). Classes of complex networks defined by role-to-role connectivity profiles. Nat. Phys..

[36] Li C, Wang L, Ji S, Chang M, Wang L, Gan Y, Liu J (2021). The ecology of the plastisphere: Microbial composition, function, assembly, and network in the freshwater and seawater ecosystems. Water Res..

[37] Ren Z, Zhang C, Li X, Ma K, Zhang Z, Feng K, Cui B (2021). Bacterial communities present distinct co-occurrence networks in sediment and water of the Thermokarst lakes in the yellow river source area. Front. Microbiol..

[38] Deng Y, Jiang YH, Yang Y, He Z, Luo F, Zhou J (2012). Molecular ecological network analyses. Bioinformatics.

[39] Singh S, Keating C, Ijaz UZ, Hassard F (2023). Molecular insights informing factors affecting low temperature anaerobic applications: Diversity, collated core microbiomes and complexity stability relationships in LCFA-fed systems. Sci. Total Environ..

[40] Tu Q, Yan Q, Deng Y, Michaletz ST, Buzzard V, Weiser MD, Waide R, Ning D, Wu L, He Z, Zhou J (2020). Biogeographic patterns of microbial co-occurrence ecological networks in six American forests. Soil Biol. Biochem..

[41] Zhang L, Delgado-Baquerizo M, Shi Y, Liu X, Yang Y, Chu H (2021). Co-existing water and sediment bacteria are driven by contrasting environmental factors across glacier-fed aquatic systems. Water Res..

[42] Yin Q, Sun Y, Li B, Feng Z, Wu G (2022). The r/K selection theory and its application in biological wastewater treatment processes. Sci. Total Environ..

[43] Chen D, Huang J, Yuan L (2019). A new function of the biocontrol bacterium *Lysobacter* enzymogenes LE16 in the mineralization of soil organic phosphorus. Plant Soil.

[44] de Vries FTD, Griffiths RI, Bailey M, Craig H, Girlanda M, Gweon HS, Hallin S, Kaisermann A, Keith AM, Kretzschmar M, Lemanceau P, Lumini E, Mason KE, Oliver A, Ostle N, Prosser JI, Thion C, Thomson B, Bardgett RD (2018). Soil bacterial networks are less stable under drought than fungal networks. Nat. Commun..

[45] Kumar T, Bhargava R, Prasad KSH, Pruthi V (2015). Evaluation of vermifiltration process using natural ingredients for effective wastewater treatment. Ecol. Eng..

[46] Ma L, Tong W, Chen H, Sun J, Wu Z, He F (2018). Quantification of N_2_O and NO emissions from a small-scale pond-ditch circulation system for rural polluted water treatment. Sci. Total Environ..

[47] Yang L, Ren Y, Zhao S, Liang X, Wang J (2016). Isolation and characterization of three heterotrophic nitrifying-aerobic denitrifying bacteria from a sequencing batch reactor. Ann. Microbiol..

[48] Zhang Y, Song C, Ji L, Liu Y, Xiao J, Cao X, Zhou Y (2018). Cause and effect of N/P ratio decline with eutrophication aggravation in shallow lakes. Sci. Total Environ..

[49] Xiao Y, Liu X, Liang Y, Niu J, Zhang X, Ma L, Hao X, Gu Y, Yin H (2016). Insights into functional genes and taxonomical/phylogenetic diversity of microbial communities in biological heap leaching system and their correlation with functions. Appl. Microbiol. Biotechnol..

[50] Li WW, Zhang HL, Sheng GP, Yu HQ (2015). Roles of extracellular polymeric substances in enhanced biological phosphorus removal process. Water Res..

[51] Li J, Sun Y, Wang X, Xu S (2020). Changes in microbial community structures under reclaimed water replenishment conditions. Int. J. Environ. Res. Publ. Health.

[52] Liu B, Yao J, Ma B, Chen Z, Zhao C, Zhu X, Li M, Cao Y, Pang W, Li H, Feng L, Mihucz VG, Duran R (2021). Microbial community profiles in soils adjacent to mining and smelting areas: Contrasting potentially toxic metals and co-occurrence patterns. Chemosphere.

