# Supplementary material for: High-proportions of tailwater discharge alter microbial community composition and assembly in receiving sediments
Source: Sci Rep. 2024 Jun 19;14:14171. doi: 10.1038/s41598-024-63859-2 (PMC11187100; doi:10.1038/s41598-024-63859-2)
Supplement: Supplementary file 1 — Supplementary Tables. [file 41598_2024_63859_MOESM1_ESM.docx]

SUPPORTING INFORMATION

**High proportions of tailwater discharge alter microbial community composition and assembly in receiving sediments^[[1]](#footnote-1)^**

Yaqian Zhou^a, b#^, Ying Lian^a#^, Li Cheng^a^, Jiaying Feng^a^, Mengling Zhou^a^, Dan Jing^a^, Weiwen Yin^a^, Heli Wang ^a, b^ *, Lei Liu ^c^ **

^a^ School of Water Resources and Environment, China University of Geosciences (Beijing), Beijing 100083, PR China.

^b^ Jiangsu Collaborative Innovation Center of Technology and Material of Water Treatment, Suzhou University of Science and Technology, Suzhou, Jiangsu 215009, China.

^c^ Key Laboratory of Tree Breeding and Cultivation of National Forestry and Grassland Administration, Research Institute of Forestry, Chinese Academy of Forestry, Beijing 100091, China.

^#^These authors contributed equally to this work.

* **Corresponding author 1:** School of Water Resources and Environment, China University of Geosciences (Beijing), Beijing 100083, PR China. *E-mail address:* [wangheli@cugb.edu.cn](mailto:wangheli@cugb.edu.cn) (H. L. Wang)

** **Corresponding author 2:** Key Laboratory of Tree Breeding and Cultivation of National Forestry and Grassland Administration, Research Institute of Forestry, Chinese Academy of Forestry, Beijing 100091, China. *E-mail address:* liulei519@caf.ac.cn (Lei Liu)

| Treatment | WF-1 | WF-2 | WF-3 | WF-4 | WF-5 |
| --- | --- | --- | --- | --- | --- |
| pH | 8.22 | 8.53 | 8.33 | 8.58 | 8.23 |
| Chemical oxygen demand  (COD, mg/L) | 9.02 | 14.04 | 14.01 | 12.00 | 10.02 |
| Phosphate (mg/L) | 0.20 | 0.19 | 0.19 | 0.18 | 0.17 |
| Ammonia nitrogen (mg/L) | 0.07 | 0.06 | 0.04 | 0.07 | 0.05 |

**Table S1. Initial water quality characteristics**

**Table S2. Alpha diversity index of the bacterial community from different treatments**

| Treatment | Sobs | Shannon | Simpson | Ace | Chao 1 | Coverage |
| --- | --- | --- | --- | --- | --- | --- |
| WF-1 | 1938.50±33.52d | 6.0979±0.0110d | 0.0056±0.0003d | 2507.64±88.47d | 2458.52±93.93d | 0.9802±0.0012d |
| WF-2 | 1993.50±122.50d | 6.1321±0.1731d | 0.0061±0.0011d | 2509.84±77.80d | 2539.27±83.11cd | 0.9805±0.0003d |
| WF-3 | 2002.03±11.22cd | 5.9661±0.0360d | 0.0076±0.0007d | 2733.70±139.66c | 2687.74±165.68bc | 0.9772±0.0022d |
| WF-4 | 2125.02±11.32bc | 6.0665±0.1082d | 0.0099±0.0041d | 2795.51±18.51c | 2762.36±14.61b | 0.9771±0.0004c |
| WF-5 | 2139.50±57.50b | 6.1480±0.1062d | 0.0066±0.0017d | 2865.48±2.61c | 2839.11±58.84b | 0.9763±0.0005c |

**Table S3. Genus of intramodule connectivity (Zi) and intermodule connectivity (Pi) of nodes in the low- (a) and high- (b) concentration clusters (Zi < 2.5 and Pi > 0.62)**

| Genus | Intramodule connectivity | Intermodule connectivity | Degree | Modularity |
| --- | --- | --- | --- | --- |
| **Low proportion** | | | | |
| *g_CL500-29_marine_group* | 0 | 1 | 1 | 5 |
| *g_norank_f__norank_o_norank_c_Subgroup_22* | 0 | 1 | 1 | 5 |
| *g_norank_f_norank_o_Chloroplast* | -1.62 | 0.75 | 6 | 2 |
| *g_Anaeromyxobacter* | -1.68 | 0.75 | 2 | 1 |
| *g_norank_f_Verrucomicrobiaceae* | -1.80 | 0.75 | 2 | 2 |
| *g_norank_f_norank_o_norank_c_SHA-26* | -1.40 | 0.75 | 2 | 1 |
| *g_Oligoflexus* | -1.80 | 0.75 | 2 | 2 |
| *g_Rubellimicrobium* | -1.41 | 0.75 | 2 | 1 |
| *g_Altererythrobacter* | -0.87 | 0.71 | 7 | 1 |
| *g_Peredibacter* | -0.87 | 0.71 | 7 | 1 |
| *g_Aquipuribacter* | -0.87 | 0.70 | 8 | 1 |
| *g_Caulobacter* | -1.20 | 0.70 | 11 | 3 |
| *g_Erysipelothrix* | -1.70 | 0.69 | 4 | 3 |
| *g_norank_f_norank_o_Vicinamibacterales* | -1.62 | 0.69 | 4 | 2 |
| *g_Lysobacter* | -1.62 | 0.69 | 4 | 2 |
| *g_Clostridium_sensu_stricto_8* | -1.62 | 0.68 | 5 | 2 |
| *g_Roseomonas* | -0.95 | 0.67 | 10 | 3 |
| *g_norank_f_norank_o_norank_c_4-29-1* | -0.45 | 0.67 | 18 | 3 |
| *g_Rhodoferax* | -1.44 | 0.64 | 6 | 2 |
| *g_norank_f_norank_o_norank_c_Gitt-GS-136* | -1.70 | 0.64 | 6 | 3 |
| *g_Thiobacillus* | -0.90 | 0.64 | 11 | 2 |
| **High proportion** | | | | |
| *g_norank_f_norank_o_WCHB1-41* | -1.20 | 0.63 | 11 | 3 |
| *g_norank_f_norank_o_norank_c_4-29-1* | -0.45 | 0.62 | 18 | 3 |

1. Abbreviations: WWTP, wastewater treatment plant; COD_Cr_, chemical oxygen demand chromium; NH_4_^+^-N, ammonium nitrogen; TP, total phosphorus; OTU, operational classification unit; NMDS, nonmetric multidimensional scale; SEM, structural equation modeling [↑](#footnote-ref-1)
